# A surrogate-based inverse design framework for targeted diameter control of electrospun nanofibers

**DOI:** 10.1038/s41598-026-40692-3

**Published:** 2026-02-25

**Authors:** Mehrab Mahdian, Ferenc Ender, Tamas Pardy

**Affiliations:** 1 Thomas Johann Seebeck Department of Electronics, Tallinn University of Technology, 12616 Tallinn, Estonia; 2 Budapest University of Technology and Economics, Department of Electron Devices, Budapest, 1111 Hungary

**Keywords:** Inverse design, Electrospinning, Optimization, Nanofibers, Engineering, Materials science

## Abstract

Electrospinning is a high-throughput technique for producing nanofibers. The diameter of such nanofibers governs key properties such as surface area, porosity, and mechanical strength. Precise diameter control is therefore crucial for applications from filtration to tissue engineering, yet optimizing processing conditions for targeted diameter fabrication typically relies on slow, costly trial-and-error experiments. This study presents a data-driven inverse-design framework that replaces traditional trial-and-error optimization with predictive modeling to achieve precise diameter control. Eleven regression models were evaluated on a dataset of 96 poly(vinyl alcohol) (PVA) experiments, with Extreme Gradient Boosting (XGBoost) emerging as the best surrogate (test $R^2 = 0.890$). SHAP analysis confirmed applied voltage and solution concentration as the most influential parameters, consistent with physical principles. In the optimization stage, Particle Swarm Optimization (PSO) achieved the highest inverse design accuracy ($R^2 = 0.991$, MAE $\approx 1.777\,\textrm{nm}$). This framework enables rapid, efficient design of nanofibers with specified properties and is readily adaptable to other materials and fabrication processes.

## Introduction

**Fig. 1:**
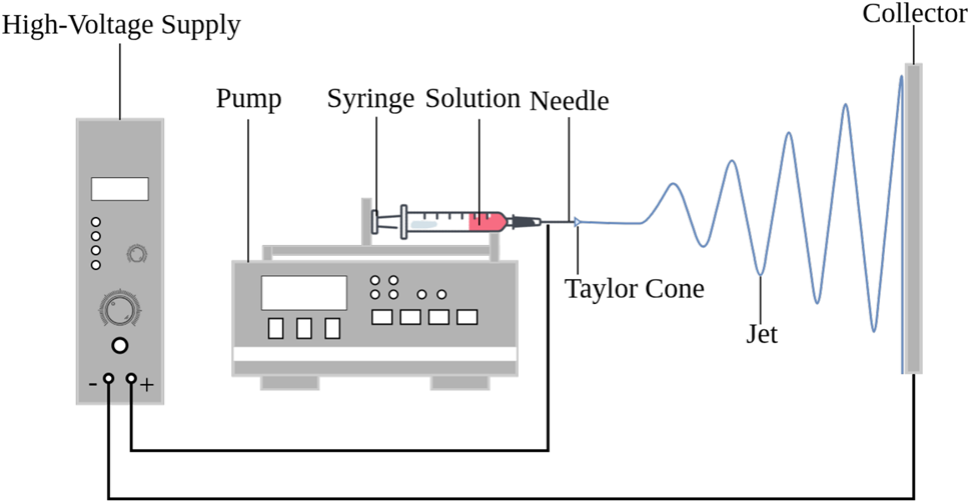
Basic electrospinning setup.

Electrospinning is a versatile and cost-effective technique for producing continuous nanofibers with diameters ranging from tens of nanometers to several micrometers^1,2^. A typical laboratory setup, shown in Fig. 1, consists of a high-voltage power supply, a syringe pump to eject a polymer solution, and a collector. The process involves the application of a strong electric field to a polymer solution, which overcomes surface tension to form a charged jet that is stretched and whipped before solidifying into nanofibers on the collector. Due to their high surface area-to-volume ratio, tunable morphology, and controllable porosity, electrospun fibers have found widespread applications in filtration^3^, tissue engineering^4^, sensors^5^, energy storage^6^, and protective textiles^7^. Despite decades of research, precise control over fiber morphology remains challenging because of the highly coupled, nonlinear relationships between process parameters and the resulting fiber morphology^8^.

This process is influenced by a combination of solution properties (e.g., viscosity, conductivity, polymer concentration, molecular weight, surface tension)^9–11^, operational settings (e.g., applied voltage, flow rate, tip-to-collector distance)^11,12^, and ambient conditions (e.g., temperature, humidity)^11,13^. Small variations in any of these parameters can significantly affect the resulting fiber morphology. Traditionally, parameter tuning has been performed through trial-and-error experiments or by following empirical qualitative guidelines derived from specific polymer-solvent systems^14^. While such approaches can yield acceptable results, they are labor-intensive, require substantial experimental resources, and do not guarantee optimal outcomes-particularly when the target fiber morphology is predetermined by application requirements. A key strategy for overcoming these limitations is a one-time, structured investment in data collection to construct a digital surrogate, which can drastically reduce the number of future experiments required to achieve new design specifications. By capturing the complex process–property relationships within a data-driven surrogate model, it becomes possible to replace iterative experimental tuning with rapid computational exploration.

Building on this strategy, data-driven modeling has emerged as a powerful tool for navigating the complex electrospinning process^15^. As a result, numerous studies have successfully employed machine learning (ML) algorithms to establish accurate *forward* models that predict fiber diameter from a set of input process parameters^16^.

In the study of Khan et al.^17^, an artificial neural network (ANN) was applied to a dataset of 100 samples to predict polyvinyl alcohol (PVA) fiber diameter, achieving a test R^2^ of 0.973 with an average absolute error of 0.06. In a similar study, Cuahuizo Huitzil et al.^18^ used a three-hidden-layer ANN to predict nanofiber diameter from PVA-based solutions and emulsions, reporting a test R^2^ of 0.98 and a prediction error of 3.79%, with viscosity identified as the most influential variable. In the study of Badaraev et al.^19^, ANN hyperparameters were optimized to predict fiber diameter and tensile strength of electrospun polycaprolactone scaffolds, outperforming traditional Box-Behnken and non-neural network methods, with polymer concentration found to be the main influencing factor. In the study of Sukpancharoen et al.^20^, Random Forest and Extra Trees Regression were applied to a dataset of approximately 430 literature data points to predict electrospun nanofiber diameters, achieving test R^2^ values of 0.947 and 0.942, with polymer concentration, applied voltage, and feed rate identified as key factors. Pervez et al.^21^ developed a locally weighted kernel partial least squares regression (LW-KPLSR) model, which outperformed other regression approaches with an R^2^ of 0.9989 and lower RMSE and MAE. Finally, in the study of Sarma et al.^22^, a novel dataset of electrospun PVDF fiber properties was used to train a multi-model machine learning framework with interpretable methods; the Gradient Boosting Regressor achieved a test R^2^ of 0.81, highlighting feed rate, solution concentration, Flory-Huggins $\chi$ parameter, and Relative Energy Difference (RED) as the main factors influencing fiber diameter, while voltage and distance showed negative correlations.

However, despite these advances, much of the existing literature has focused primarily on *forward modeling*, which predicts fiber properties from known process parameters. While effective for understanding process–property relationships, this approach typically requires a separate, iterative inversion step to identify parameters corresponding to a desired output. Such inversion procedures can be computationally expensive and may not always converge to experimentally feasible solutions^23^. As a result, forward modeling alone does not fully enable a predictive and prescriptive framework for electrospinning process design.

Inverse design offers a powerful alternative strategy: instead of predicting fiber properties from known process parameters, the goal is to directly determine the optimal set of experimentally feasible parameters required to achieve a desired fiber diameter. In the field of materials science and manufacturing, this inverse approach has been increasingly enabled by machine learning (ML) and surrogate modeling. These data-driven models can capture the complex, nonlinear relationships from experimental data and allow for efficient, global exploration of the high-dimensional parameter space through sophisticated optimization algorithms^24^. Indeed, such approaches have been successfully demonstrated across various fields, including the design of photonic meta-surfaces^25,26^, the optimization of additive manufacturing processes^27^, and the development of thin-film materials^28^. Moreover, the study of McDonald et al.^29^, highlighted the potential of machine learning in accelerating the design of medical-grade polymers by bypassing trial-and-error synthesis, despite challenges from limited standardized data on degradation time and biocompatibility, thereby emphasizing the promise of data-driven inverse design approaches. Nonetheless, as identified in the prior review, the adoption of a true inverse design framework in electrospinning has been limited. Most ML applications remain focused on forward prediction, and the few existing inverse attempts often neglect critical practical constraints, failing to guarantee that the identified parameters are robust and experimentally feasible. This gap highlights a significant opportunity to develop an inverse design methodology specifically tailored for the electrospinning process.

This work introduces a systematic data-driven inverse-design framework aimed at precise nanofiber diameter control. Using poly(vinyl alcohol) (PVA) nanofibers as a model system, we leverage a high-quality dataset obtained via response surface methodology (RSM) with a four-factor, four-level design, comprising 96 systematically varied experimental trials. Eleven regression algorithms are evaluated to identify an accurate surrogate model for fiber-diameter prediction, with Extreme Gradient Boosting (XGBoost) emerging as the top performer. This surrogate model is then integrated with a range of optimization techniques–including brute-force grid search, random search, Simulated Annealing (SA), Genetic Algorithm (GA), Differential Evolution (DE), Particle Swarm Optimization (PSO), and Bayesian Optimization (BO)–to determine process parameters that achieve target fiber diameters.

The key contributions of this work are threefold: (i) we establish a systematic surrogate-based inverse-design framework tailored for electrospinning, (ii) we rigorously benchmark seven optimization strategies and demonstrate the superior robustness and accuracy of PSO when coupled with an XGBoost surrogate, and (iii) we provide a fully reproducible, interpretable workflow that transforms electrospinning from a trial-and-error process into a predictive, prescriptive design task.

## Materials and methods

### Dataset description

The experimental data used in this study were sourced from the work of Ziabari et al.^30^. This dataset was selected for its systematic investigation of the effect of four key operating parameters, applied voltage, solution concentration, feed rate, and tip-to-collector distance on the resulting average fiber diameter and diameter variation. In the original experiments, poly(vinyl alcohol) (PVA) with a molecular weight of 72,000 g/mol was dissolved in distilled water and electrospun using a horizontal setup under ambient laboratory conditions, where temperature, humidity, and air pressure were kept constant.

The dataset was constructed using a Response Surface Methodology (RSM) framework, involving four factors, each sampled at four levels, resulting in a total of 96 unique experimental trials. The original study selected these parameters as the most influential, with their ranges carefully chosen through preliminary experiments to ensure the production of dry, bead-free, and continuous fibers^30^. Under the constant experimental conditions (fixed polymer molecular weight and temperature), solution concentration was treated as a direct proxy for solution viscosity^30^. This made the explicit measurement of viscosity redundant and resulted in a parsimonious yet physically comprehensive feature set. The factors and their corresponding values for the coded levels used in the experimental design are detailed in Table 1.

**Fig. 2:**
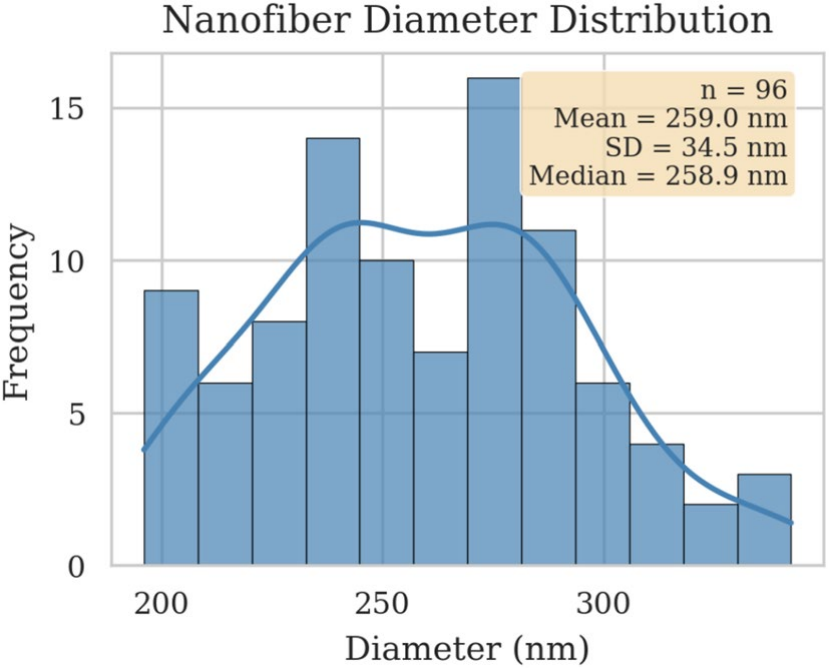
Distribution of measured nanofiber diameters across the 96 experimental trials in the dataset. The histogram illustrates the range and frequency of the target output variable.

The fiber diameters, which served as the target output variable for our models, were measured from scanning electron microscopy (SEM) micrographs. The distribution of these measured diameters is shown in Fig. 2, with values ranging approximately from 200 nm to 350 nm.

**Table 1:** Experimental factors, their actual values for the coded levels, and the number of observations at each level. The design ensures a balanced exploration of the parameter space.

Factor	− 1	− 0.5	0	1
Values				
Solution concentration (wt%)	8	9	10	12
Tip-to-collector distance (cm)	10	12.5	15	20
Applied voltage (kV)	15	20	22.5	25
Feed rate (mL/h)	0.20	0.25	0.30	0.40
Frequency				
Solution concentration	27	8	34	27
Tip-to-collector distance	27	8	27	34
Applied voltage	34	8	27	27
Feed rate	27	8	34	27

The dataset’s comprehensive structure was leveraged to maximize the information available for training our models. While a perfectly symmetric, full-factorial design is often experimentally infeasible due to cost and time constraints, this dataset, which utilizes a multi-level structure with a non-uniform distribution of observations, is representative of a complex, realistically structured experimental space. Utilizing this complete set of trials provided a robust foundation for the subsequent inverse design optimization, ensuring the resulting models could effectively learn complex, non-linear relationships and demonstrating a robustness essential for broad applicability.

### Machine learning framework

The development of the inverse design framework was conducted in two main phases. The First Phase focused on the selection, hyperparameter tuning, and cross-validated evaluation of the surrogate model. The Second Phase was dedicated to the implementation, hyperparameter tuning, and rigorous evaluation of the optimization algorithms.

#### Development dataset formulation

As the initial step to ensure predictive accuracy and generalizability, the 96-trial dataset^30^ was divided into an 80% development set and a 20% held-out test set. The development set was used for all training and hyperparameter tuning processes (e.g., via nested cross-validation), while the reserved test set was used solely for the final, unbiased validation of the surrogate model and the complete inverse design framework.

To ensure full reproducibility across both the surrogate modeling and optimization stages, all stochastic components of the framework, including data splitting, model initialization, cross-validation procedures, and optimizer initialization, were controlled using a fixed random seed of 42.

#### Surrogate modeling

Surrogate modeling, also referred to as meta-modeling, response surface modeling, or emulation, is a technique for constructing a simplified, data-driven, and computationally inexpensive approximation of a complex system or process^31–33^. The core premise is to replace a resource-intensive function, be it a high-fidelity simulation or a physical experiment with a cost-effective statistical model, thereby enabling rapid exploration and optimization^34^. In the context of resource-intensive physical experiments like electrospinning, this approach addresses the core challenge outlined in the introduction: instead of conducting a costly and time-consuming campaign of trial-and-error experiments, a surrogate model is trained on a limited set of carefully designed input-output pairs to emulate the process’s behavior.

**Fig. 3:**
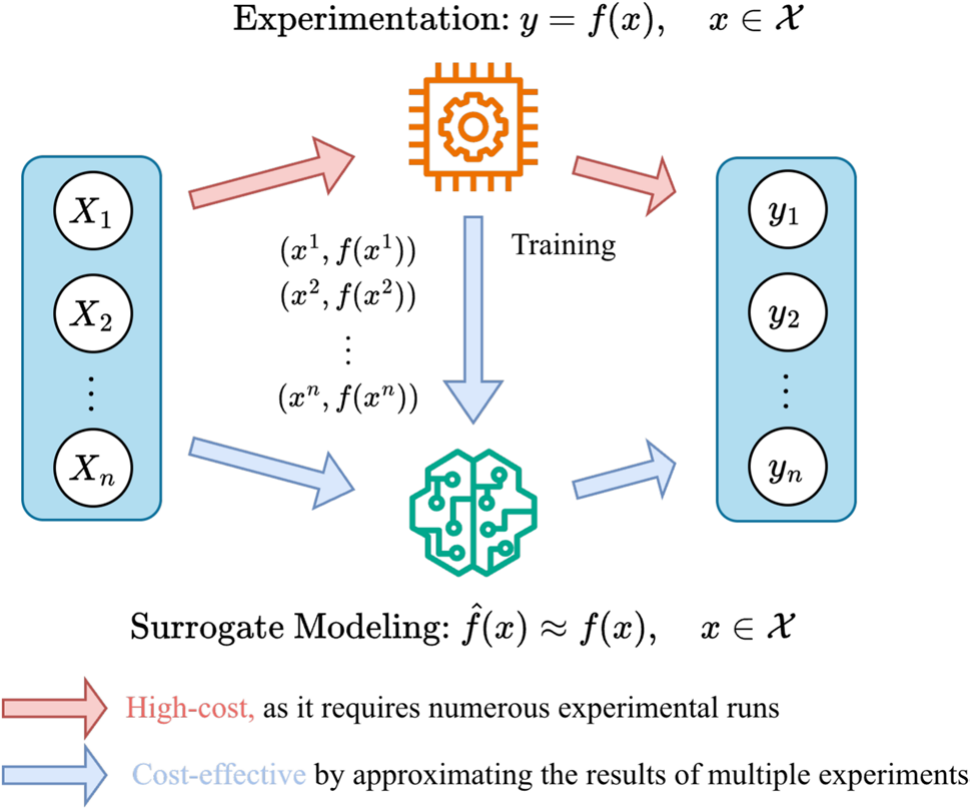
Concept of surrogate modeling for experimental processes. The function $y = f(x)$ represents the unknown, complex physical process (the experiment). A cost-effective surrogate model is trained on empirical input-output data $(X_n, y_n)$ obtained from physical experiments to approximate the system’s behavior, enabling rapid prediction and optimization.

As illustrated in Fig. 3, the surrogate is built from high-fidelity data-in this case, empirical measurements from actual laboratory trials. Once trained, it provides predictions that are comparable to those from new experiments, while dramatically reducing the experimental effort, cost, and time required for exploration and optimization.

Surrogate models are widely used in applications such as design optimization, uncertainty quantification^35^, and sensitivity analysis^36^. They are particularly valuable in fields like materials science and manufacturing, where they enable efficient navigation of complex parameter spaces^37,38^. This makes them an ideal foundation for the inverse design framework developed in this work.

#### Surrogate model selection

**Fig. 4:**
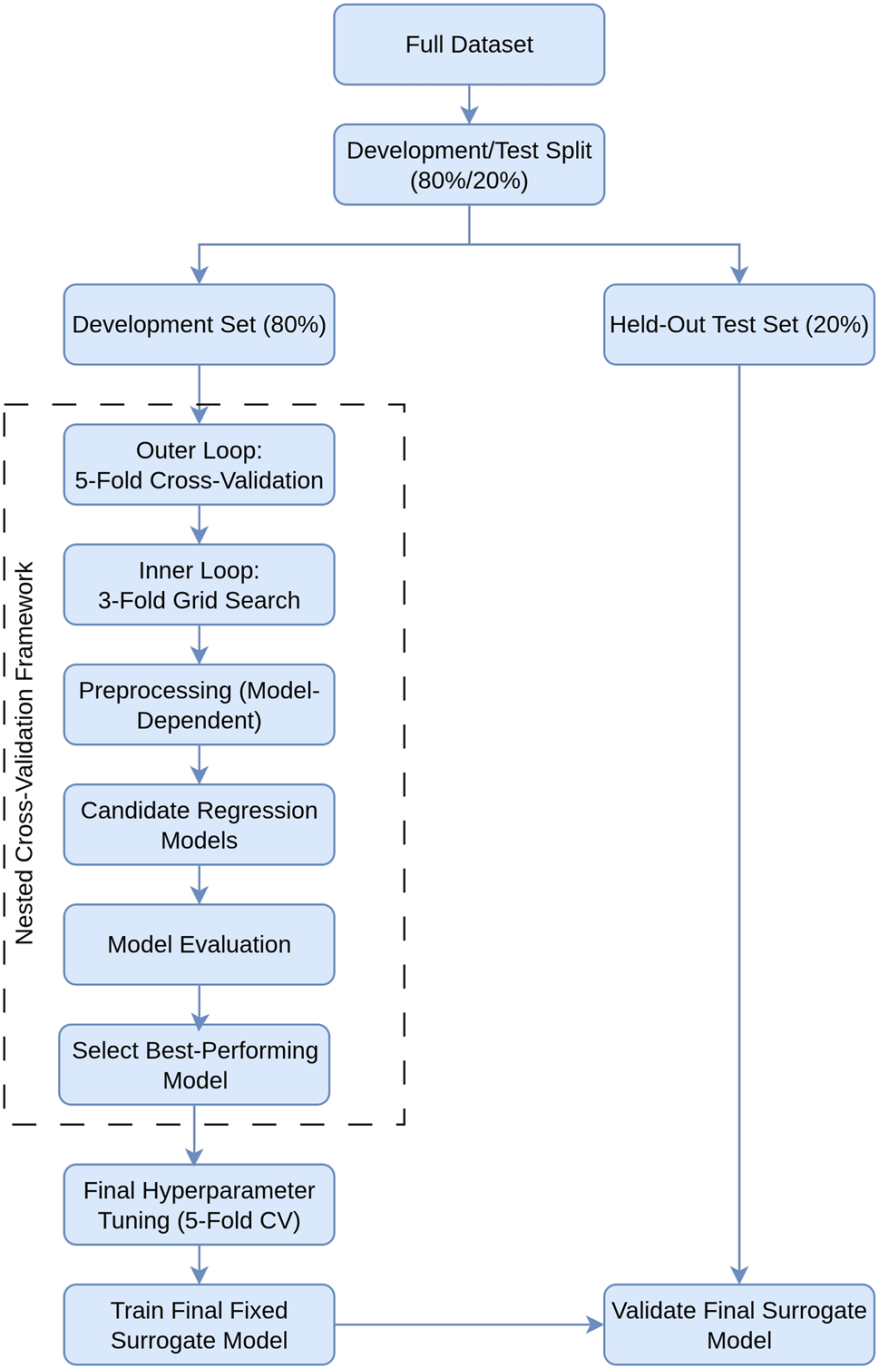
Machine learning workflow for surrogate model selection and evaluation.

Following the machine learning workflow illustrated in Fig. 4, surrogate model selection was conducted through a structured pipeline consisting of data formulation and preprocessing, model training, cross-validated evaluation, and comparative model selection.

The mean fiber diameter was defined as the response variable, while the four electrospinning process parameters–applied voltage, solution concentration, feed rate, and tip-to-collector distance–were used as input features. The dataset was divided into an 80% development set and a 20% held-out test set. For regression models sensitive to feature scale, a preprocessing pipeline was applied to standardize the input features to zero mean and unit variance, ensuring that variables with larger numerical ranges did not disproportionately influence model training.

A total of eleven regression algorithms were evaluated, encompassing a diverse range of modeling paradigms:

- *Linear models:* Linear Regression (LR), Ridge, Lasso, Elastic Net (EN)
- *Tree-based ensembles:* Decision Tree (DT), Random Forest (RF), Gradient Boosting (GB), AdaBoost (AB), XGBoost (XGB)
- *Other methods:* K-Nearest Neighbors (KNN), Multi-Layer Perceptron (MLP)

Model hyperparameters were optimized and performance was estimated using a nested cross-validation framework applied to the 80% development set. Specifically, an outer 5-fold cross-validation loop was employed for unbiased performance estimation, while an inner 3-fold grid search^39^ was used for exhaustive hyperparameter tuning. This procedure ensured fair comparison across models while mitigating overfitting.

Model performance was assessed using three complementary metrics: root mean squared error (RMSE), mean absolute error (MAE), and the coefficient of determination ($R^2$)^39,40^.

Based on cross-validated performance across these metrics, the best-performing algorithm was selected as the surrogate model. All candidate models were trained and hyperparameter-tuned exclusively on the 80% development set, ensuring a consistent and fair comparison. The selected model was then retrained using the optimal configuration on the full development set and subsequently evaluated on the independent 20% held-out test set to assess predictive robustness and generalization. The finalized surrogate model was saved for use in the subsequent inverse design and optimization phase.

*Evaluation metrics:*

- *Root mean squared error (RMSE)* Quantifies the average magnitude of prediction errors, penalizing larger deviations more heavily:
  1 $$\begin{aligned} \textrm{RMSE} = \sqrt{\frac{1}{n} \sum _{i=1}^{n} (y_i - \hat{y}_i)^2} \end{aligned}$$
  where $y_i$ and $\hat{y}_i$ represent the observed and predicted values for sample $i$, and $n$ denotes the number of samples.
- *Mean absolute error (MAE)* Captures the average magnitude of prediction errors, disregarding their direction:
  2 $$\begin{aligned} \textrm{MAE} = \frac{1}{n} \sum _{i=1}^{n} |y_i - \hat{y}_i| \end{aligned}$$
- *Coefficient of determination *($R^2$) Measures the proportion of variance in the target variable explained by the model:
  3 $$\begin{aligned} R^2 = 1 - \frac{\sum _{i=1}^{n} (y_i - \hat{y}_i)^2}{\sum _{i=1}^{n} (y_i - \bar{y})^2} \end{aligned}$$
  where $\bar{y}$ is the mean of observed values. An $R^2$ value close to 1 indicates that the model captures most of the variability in the data.

Evaluation metrics are summarized using the “mean ± standard deviation” format, which conveys both the central tendency of the metric and its variability across repeated evaluations. The mean reflects the typical value of the metric, while the standard deviation characterizes the spread of the individual estimates and therefore the stability of the model or optimizer across repetitions.

In addition to these summary statistics, 95% confidence intervals (CIs) are provided to quantify the uncertainty in the estimated performance metrics. A confidence interval represents a range of plausible values for the true metric under repeated sampling from the same data-generating process; narrower intervals indicate higher stability, whereas wider intervals reflect greater uncertainty.

For reproducibility, the entire machine learning framework was implemented in Python 3.10. The core packages utilized for the surrogate modeling and evaluation include: numpy, pandas, scikit-learn, and xgboost.

#### Model interpretability using SHAP

To enhance the transparency and trustworthiness of the selected surrogate model, we employed SHapley Additive exPlanations (SHAP), a unified framework based on cooperative game theory that interprets the output of machine learning models^41^. This analysis quantifies the contribution of each input parameter (applied voltage, solution concentration, feed rate, and tip-to-collector distance) to the model’s predictions, identifying the most influential factors and their directional effect on the predicted nanofiber diameter.

To generate a robust, consolidated, and bias-free interpretation across the entire experimentally accessible domain, SHAP values were computed by applying a standard 5-fold cross-validation scheme across the entire original 96-trial dataset. Within this scheme, for each fold, a new XGBoost model was retrained from scratch using the optimal hyperparameters determined in the preceding tuning phase. The $\texttt {TreeExplainer}$ class from the SHAP Python library then calculated exact Shapley values for the corresponding validation subset of that fold. These values, representing the average marginal contribution of each feature, were aggregated across all five folds to provide a comprehensive view of global feature importance across the entire dataset.

This approach enables robust global interpretability, ranking overall feature importance across the parameter space, and local interpretability, explaining individual predictions by showing how each feature influenced the output.

#### Optimization with surrogate modeling

Following the model comparison, the Extreme Gradient Boosting (XGBoost) algorithm was chosen as the optimal surrogate model due to its superior predictive performance, as indicated by the highest consistent $R^2$ score. This high-fidelity surrogate was then used within an inverse design framework. The core objective of this framework is to identify combinations of the four process parameters that yield a predicted fiber diameter as close as possible to a pre-specified target value, $d_{\textrm{target}}$.

The optimization task was formally defined as minimizing the squared error between the surrogate model’s prediction and the target diameter:

4 $$\begin{aligned} \min _{\textbf{x} \in \mathscr {X}} \left( f_{\textrm{surrogate}}(\textbf{x}) - d_{\textrm{target}} \right) ^2 \end{aligned}$$

where $\textbf{x}$ is a vector representing a set of process parameters within the feasible domain $\mathscr {X}$, and $f_{\textrm{surrogate}}$ is the trained XGBoost model. The feasible domain, $\mathscr {X}$, is defined by the minimum and maximum actual values of each factor used in the original experimental space (Table 1), which represent the optimal working ranges that resulted in a stable electrospinning process. Specifically, these boundaries are: Solution Concentration (8–12 wt%), Applied Voltage (15–25 kV), Tip-to-Collector Distance (10–20 cm), and Feed Rate (0.20–0.40 mL/h).

**Fig. 5:**
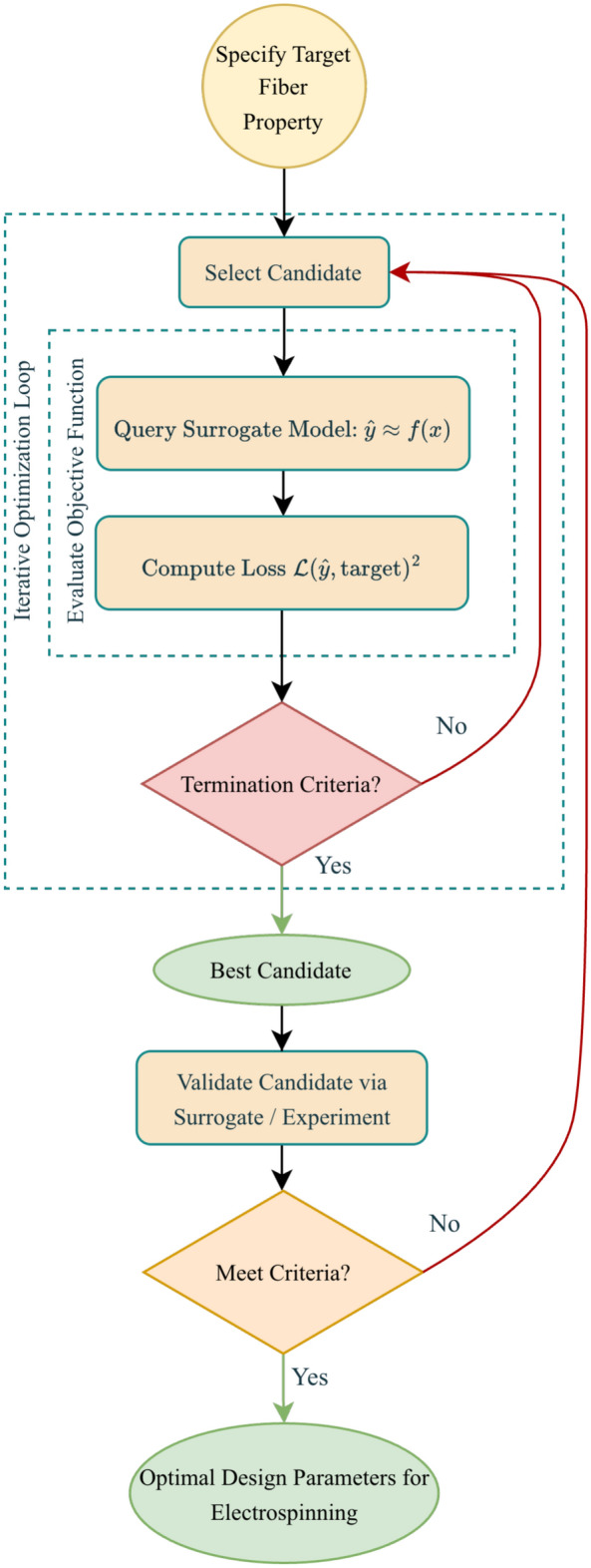
Workflow for surrogate-based inverse design. An optimization algorithm iteratively queries the trained surrogate model to find parameter sets $\textbf{x}$ that minimize the error between the predicted fiber diameter $\hat{y}=f_{surrogate}(\textbf{x})$ and the target diameter $d_{\textrm{target}}$.

The workflow for this inverse design, illustrated in Fig. 5, establishes a systematic, closed-loop process. At its core, the pre-trained surrogate model, $f_{surrogate}(\textbf{x})$, functions as a digital twin, emulating the relationship between process parameters $\textbf{x}$ and fiber diameter $\textbf{y}$. The user specifies a target fiber diameter, $d_{target}$, which defines the optimization goal. The actual objective function (or loss function) being minimized is the squarred error, $\mathscr {L}$, between the diameter predicted by the surrogate model, $\hat{y}=f_{surrogate}(\textbf{x})$, and the target diameter, $d_{target}$. The optimization algorithm then iteratively generates candidate parameter sets and queries the surrogate model to obtain a predicted diameter for each.

The specific search strategy varies by algorithm, but all aim to balance exploration of the global parameter space with exploitation of promising regions. Seven distinct optimization algorithms, each with a unique mechanistic approach to this trade-off, were implemented and compared for solving this inverse problem.

- *Deterministic/Exhaustive search* Methods that systematically evaluate all candidate points from a predefined set, guaranteeing the best solution on that grid.
  - *Brute-force grid search:* Evaluates every combination of parameters on a fixed discretized grid. For *N* parameters, each with *n* possible values, this results in $N^n$ total combinations, illustrating how quickly computational cost grows with problem size.
- *Stochastic search* Approaches that rely on random sampling to generate new candidate points.
  - *Random search:* Selects parameter sets uniformly at random from the search space. In our study, we evaluated 50 randomly selected parameter combinations.
- *Single-solution metaheuristic* Heuristics that maintain and iteratively refine a single candidate solution, using probabilistic moves to escape local minima and gradually focus on exploitation.
  - *Simulated annealing (SA):* Models the physical annealing process. It starts with a high “temperature” *T*, allowing probabilistic acceptance of worse solutions (uphill moves) to escape local minima. The temperature cools over time according to a schedule (e.g., $T_{k+1} = \alpha T_k$), gradually reducing the acceptance probability of worse solutions so the search converges toward a (hopefully global) minimum.
- *Population-based metaheuristics* Algorithms that evolve or coordinate a population of candidate solutions. Information sharing among individuals enables broad exploration and local refinement.
  - *Genetic algorithm (GA):* Evolves a population through biologically-inspired operators. Selection exploits fitter individuals, while crossover (e.g., $\vec {c} = \beta \vec {p_1} + (1-\beta )\vec {p_2}$) and mutation (e.g., $x_{\text {new}} = x_{\text {old}} + \mathscr {N}(0, \sigma )$) promote exploration and diversity to guide the search.
  - *Differential evolution (DE):* Creates new candidates through a distinctive mutation strategy. For a target vector $\vec {x_i}$, a mutant vector is generated via $\vec {v} = \vec {x_{r1}} + F \cdot (\vec {x_{r2}} - \vec {x_{r3}})$, where *F* is a mutation factor and *r*1, *r*2, *r*3 are distinct indices. This leverages the population’s diversity to explore the space efficiently.
  - *Particle swarm optimization (PSO):* Models a swarm of particles where each particle *i* updates its position $\vec {x_i}$ and velocity $\vec {v_i}$ based on its own best solution ($\vec {p}_{\text {best},i}$) and the swarm’s global best ($\vec {g}_{\text {best}}$): $\vec {v_i}(t+1) = w \cdot \vec {v_i}(t) + c_1 r_1 (\vec {p}_{\text {best},i} - \vec {x_i}(t)) + c_2 r_2 (\vec {g}_{\text {best}} - \vec {x_i}(t)); \quad \vec {x_i}(t+1) = \vec {x_i}(t) + \vec {v_i}(t+1)$. This balances personal experience and social learning.
- *Model-based/Surrogate optimization* Techniques that construct a probabilistic surrogate model of the objective function and use it to select new evaluations that balance exploration and exploitation.
  - *Bayesian optimization (BO):* Builds a probabilistic surrogate model, typically a Gaussian Process (GP), $f(\textbf{x}) \sim \mathscr{G}\mathscr{P}(m(\textbf{x}), k(\textbf{x}, \textbf{x}'))$, to approximate the objective function. It then uses an acquisition function, which is maximized to select the next query point: $\textbf{x}_{\text {next}} = \arg \max _{\textbf{x}} \alpha (\textbf{x})$. This mathematically balances exploring uncertain regions and exploiting promising ones. A key distinction in this framework is the role of two separate surrogate models: the pre-trained surrogate (i.e., XGBoost) provides the raw prediction ($\hat{y}$) used to compute the error, while the internal Gaussian Process (GP) surrogate models the objective function itself (the squared error $|\hat{y} - d_{\text {target}}|^2$), consistent with the objective function used by all optimizers in the implementation. The GP surrogate guides the optimization search by predicting the objective value at unexplored points, but it is always fitted on the errors produced by the pre-trained surrogate.

These structured search strategies provide a non-random strategy for navigating the complex parameter space. The iterative process continues until a termination criterion is satisfied, such as convergence to an optimal solution (e.g., via early stopping) or the completion of a maximum number of iterations. The final output is an optimized set of process parameters predicted to yield the desired fiber diameter. The best candidate parameters identified through this process can be validated against the surrogate model or through physical experimentation, ensuring practical feasibility and accuracy before final selection. This integrated framework enables efficient, data-driven inverse design, significantly reducing reliance on costly and time-consuming experimental trials.

Because the optimization algorithms are stochastic, each inverse design task was repeated five times using different random initializations, and performance metrics were aggregated across these independent replicas to ensure robustness.

For reproducibility, the optimization algorithms were implemented using a combination of specialized and custom modules. The Bayesian Optimization (BO) technique utilized the scikit-optimize package. The remaining metaheuristic algorithms-Particle Swarm Optimization (PSO), Genetic Algorithm (GA), Differential Evolution (DE), and Simulated Annealing (SA) were implemented as custom, standalone Python modules developed specifically for this inverse design task. All code, dependencies, and version information are available in the public repository.

#### Hyperparameter tuning of optimization algorithms

To ensure optimal performance for the inverse design task, all optimization algorithms with tunable hyper-parameters–Simulated Annealing (SA), Genetic Algorithm (GA), Differential Evolution (DE), Particle Swarm Optimization (PSO), and Bayesian Optimization (BO)–underwent systematic hyperparameter tuning. A grid search approach^39^ was employed, where each combination of predefined hyperparameter values was evaluated using the surrogate model.

For each hyperparameter configuration, candidate parameter sets were generated, and their search performance was assessed across the entire 80% development set. It is important to note that these algorithms are fundamentally probabilistic search models that explore the objective landscape defined by the surrogate model. Even BO, with its internal Gaussian Process, is used to optimize the search strategy rather than learn a predictive model. As a result, their tuning focuses on optimizing search efficiency and convergence rather than generalization error. The combination that yielded the best performance (minimum mean absolute error) on the development set was selected as the final configuration for the corresponding optimization algorithm.

Table 2 summarizes the full hyperparameter grids explored for all algorithms.

**Table 2:** Hyperparameter grids for each optimization algorithm.

Optimizer	Hyperparameters
Simulated annealing (SA)	Temp: [50,100,150,200,300], Cooling: [0.80,0.85,0.90,0.95,0.98]
	Iterations per temp: [20,50,100], Step size: [0.1,0.2]
Genetic algorithm (GA)	Population: [20,30,40,50], Generations: [50,100]
	Crossover: [0.6,0.8,1.0], Mutation rate: [0.01,0.05,0.1], Mutation scale: [0.05,0.1,0.2]
Differential evolution (DE)	Population: [30,50,70], Generations: [80,110,140,170]
	Crossover: [0.6,0.75,0.9], Mutation factor: [0.3,0.45,0.6,0.75]
Particle swarm optimization (PSO)	Particles: [20,30,40,50], $w_{\max }/w_{\min }: [0.8,0.9]/[0.3,0.4]$
	$c_1/c_2: [1.5,2.0]$, Max velocity: [0.1,0.2,0.3]
Bayesian optimization (BO)	$n_{\text {init}}: [5,10,15]$, $n_{\text {iter}}: [30,50,70]$
	Acquisition: [EI, PI, UCB], $\kappa : [1.5,2.5]$, $\xi : [0.005,0.01,0.02]$
Random search	Not applicable; samples random points without tunable parameters.
Brute-force grid search	Not applicable; grid defined by the problem’s parameter space.

## Results

### Performance of regression models

The predictive performance of the eleven regression models is summarized in Table 3. The results were obtained from the five outer folds of the nested cross-validation performed on the 80% development set, providing an unbiased estimate of the models’ generalization error. Following model selection and final hyperparameter tuning, the fixed models were evaluated on the 20% held-out test set, with results summarized in Table 4.

Nested cross-validation performance on the 80% development set (Table 3) revealed that XGBoost consistently outperformed all other models, achieving the lowest RMSE ($10.12 \pm 2.02$ nm) and MAE ($7.95 \pm 1.29$ nm), alongside a high $R^{2}$ of $0.89 \pm 0.05$. This superior performance was calculated across the 5 outer folds of the nested cross-validation, demonstrating its robust generalization capability.

Gradient Boosting followed closely, with marginally higher prediction errors (RMSE = $11.60 \pm 1.92$ nm, MAE = $9.08 \pm 1.28$ nm, $R^{2} = 0.86 \pm 0.04$) . Random Forest exhibited a noticeable increase in testing errors and a reduced $R^{2}$ ($0.83 \pm 0.08$), confirming a degree of overfitting . The remaining models, including KNN, Decision Tree, AdaBoost, and all linear regressions, performed poorly, with $R^{2}$ values falling at or below 0.73, highlighting their inability to generalize effectively .

Following the selection of the optimal model and the final determination of the best hyperparameters for all candidates, each fully optimized model was evaluated on the 20% held-out test set (Table 4) to provide the ultimate, unbiased measure of predictive power.

The final evaluation showed that XGBoost, which was selected as the optimal model based on the nested CV performance, maintained its strong predictive ability, yielding an $R^{2}$ of 0.890, an MAE of 11.26 nm, and an RMSE of 13.56 nm. This confirms the robustness and high predictive accuracy of the final surrogate model when presented with completely unseen data. Similarly, all other optimized models were also evaluated, with Gradient Boosting ($R^{2}$ of 0.868) and MLP Regressor ($R^{2}$ of 0.849) demonstrating competitive performance on the held-out set , reinforcing that ensemble and non-linear methods are best suited for capturing the complex electrospinning physics.

**Table 3:** Performance of regression models comparison using cross-validation on the 80% development set. Values are reported as mean ± standard deviation (95% CI) across the outer folds.

Model	Testing performance		
	RMSE	MAE	$R^2$
XGBoost	10.12 ± 2.02 [7.62, 12.63]	7.95 ± 1.29 [6.35, 9.54]	0.89 ± 0.05 [0.83, 0.95]
Gradient boosting	11.60 ± 1.92 [9.21, 13.99]	9.08 ± 1.28 [7.49, 10.66]	0.86 ± 0.04 [0.82, 0.91]
Random forest	12.78 ± 3.32 [8.66, 16.90]	10.19 ± 2.65 [6.89, 13.48]	0.83 ± 0.08 [0.73, 0.93]
AdaBoost	13.73 ± 3.76 [9.07, 18.40]	10.96 ± 3.48 [6.64, 15.28]	0.81 ± 0.07 [0.72, 0.90]
Decision tree	16.51 ± 1.59 [14.54, 18.49]	12.67 ± 2.42 [9.66, 15.68]	0.73 ± 0.07 [0.64, 0.81]
KNN	18.02 ± 2.06 [15.46, 20.58]	15.17 ± 1.84 [12.88, 17.45]	0.68 ± 0.04 [0.62, 0.73]
Linear regression	20.92 ± 4.47 [15.36, 26.47]	17.82 ± 3.95 [12.92, 22.73]	0.56 ± 0.14 [0.39, 0.74]
Lasso regression	20.78 ± 4.58 [15.09, 26.47]	17.67 ± 4.32 [12.31, 23.03]	0.57 ± 0.13 [0.41, 0.73]
Ridge regression	21.10 ± 4.73 [15.22, 26.97]	17.87 ± 4.39 [12.42, 23.32]	0.55 ± 0.14 [0.38, 0.73]
ElasticNet	21.26 ± 4.90 [15.19, 27.34]	18.18 ± 4.52 [12.57, 23.79]	0.55 ± 0.13 [0.39, 0.72]
MLP regressor	30.37 ± 11.23 [16.42, 44.32]	23.61 ± 8.10 [13.56, 33.67]	− 0.06 ± 0.87 [-1.13, 1.02]

**Table 4:** Final testing performance of regression models on the 20% held-out test set.

Model	Testing performance		
	RMSE	MAE	$R^2$
XGBoost	13.56	11.16	0.890
Gradient boosting	14.84	12.17	0.868
MLP regressor	15.88	12.85	0.849
Decision tree	16.39	13.53	0.839
Random forest	20.07	15.00	0.758
KNN	20.32	14.63	0.752
Linear regression	20.52	15.64	0.748
Lasso regression	20.53	15.64	0.747
ElasticNet	20.63	15.65	0.745
Ridge regression	20.68	15.65	0.744
AdaBoost	20.78	17.52	0.741

#### Surrogate model accuracy and predictive power

**Fig. 6:**
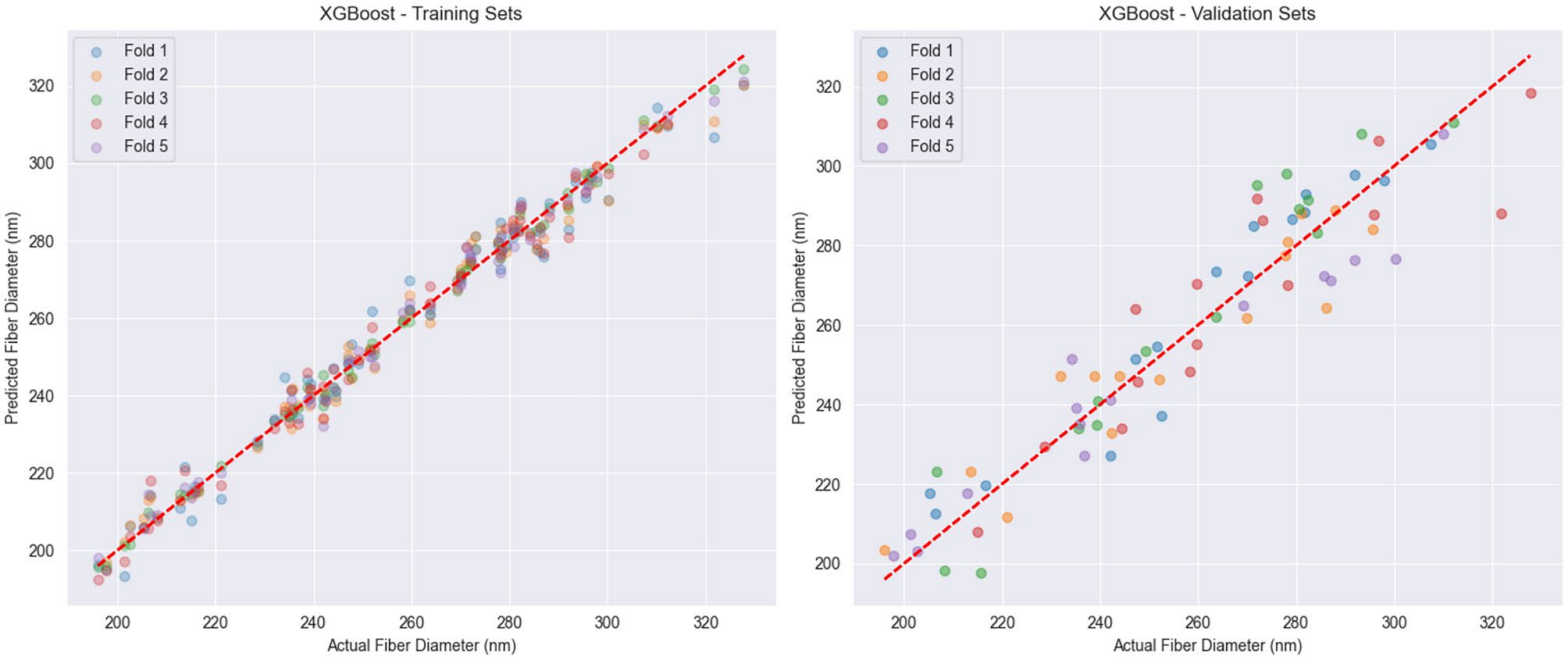
Parity plots of XGBoost predictions versus measured fiber diameters evaluated during cross-validation on the 80% development set. The left plot shows predictions for the training folds, while the right plot shows predictions for the corresponding validation folds. The red dashed line represents the ideal prediction (R^2^ = 1).

The XGBoost algorithm, identified as the top-performing model, was selected as the surrogate for the inverse design framework. Its predictive accuracy and generalization capability were further assessed through parity analysis across the 5 outer folds of the nested cross-validation (on the 80% development set), as shown in Fig. 6

On the training data (Fig. 6, left), the model predictions align closely with the ideal parity line, with a tightly clustered distribution of points. This indicates excellent fit with minimal bias and high capacity to capture nonlinear relationships within the training domain .

Predictions on the validation sets (Fig. 6, right) also show strong agreement with experimental measurements ($R^{2} = 0.896 \pm 0.05$), confirming robust generalization . A slight increase in dispersion is observed, particularly at larger fiber diameters ($>300\,\textrm{nm}$), which can be attributed to the reduced density of training samples in this range and the inherent variability of the electrospinning process at higher parameter values .

**Fig. 7:**
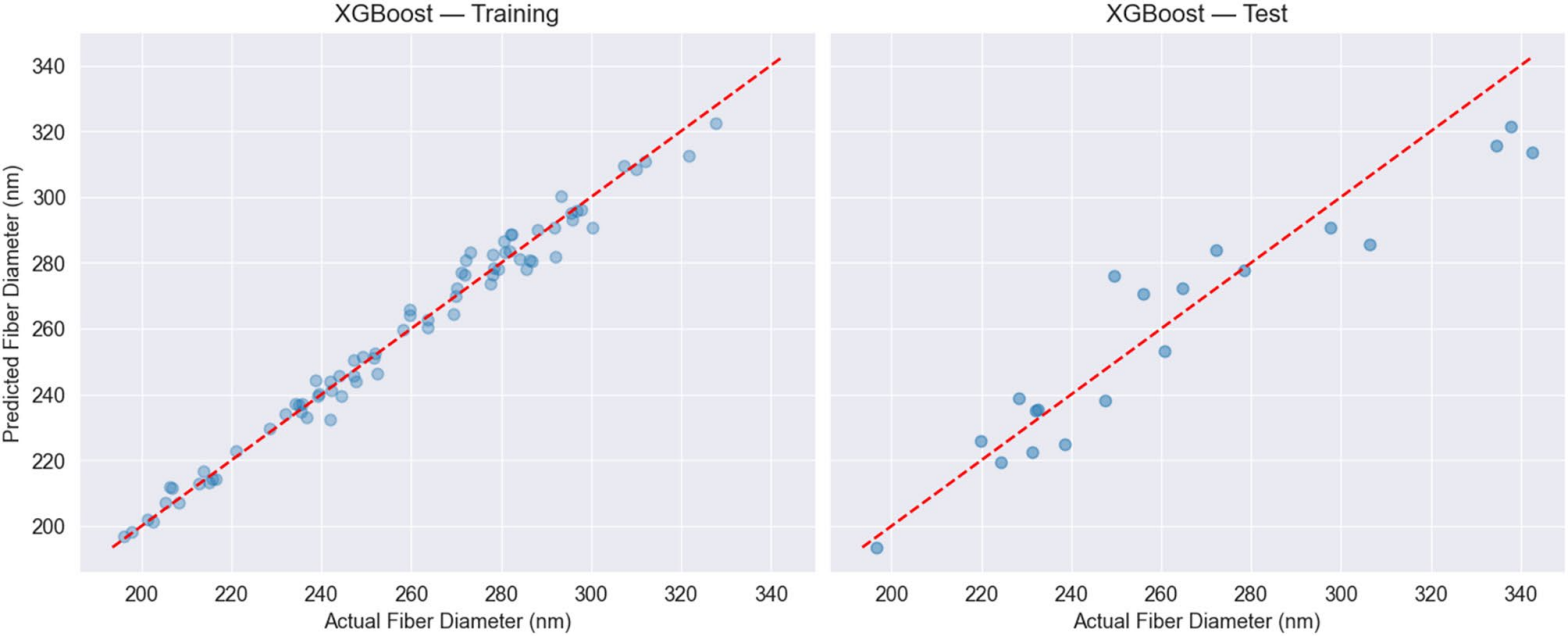
Parity plots of the final XGBoost model performance. The left plot shows predictions versus measured fiber diameters for the 80% development set used for final training, while the right plot shows predictions evaluated on the unseen 20% held-out test set. The red dashed line represents the ideal prediction (R^2^ = 1).

The consistency across cross-validation folds supports the robust approximation of the input–output relationships in electrospinning . Its performance was confirmed by a final parity analysis against the 20% held-out test set, as shown in Fig. 7. Despite minor deviations at the extremes, the model maintains high predictive accuracy across the majority of the diameter range. This fixed, high-fidelity model is thus considered a reliable surrogate for the subsequent optimization task.

#### SHAP-based model interpretability

**Fig. 8:**
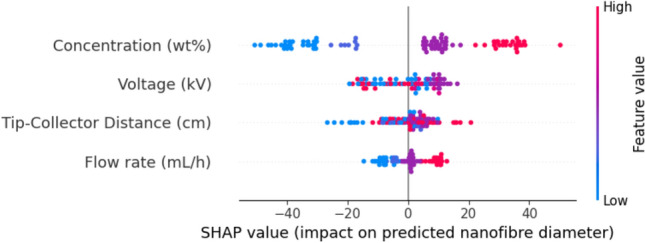
SHAP summary plot illustrating the influence of each input feature on the XGBoost surrogate model’s predictions. The x-axis represents the SHAP value (impact on model output), and the color indicates the feature value (blue = low, red = high).

Figure 8 shows a SHAP summary plot illustrating the global feature importance. Each point in the plot represents a Shapley value for a particular feature for a single prediction. The x-axis indicates the impact of the feature value on the model’s prediction, while the color corresponds to the original feature value, ranging from low (blue) to high (red).

Applied voltage and solution concentration are the most influential features affecting the model predictions. Higher applied voltage values (red points) correspond to lower SHAP values, indicating a strong negative relationship where increasing voltage tends to decrease the predicted fiber diameter. This aligns with the electrohydrodynamic principle that a stronger electric field imposes greater stretching forces on the polymer jet, leading to thinner fibers. In contrast, higher solution concentration values (red points) are associated with higher SHAP values, suggesting that increasing concentration leads to larger predicted fiber diameters. This reflects the role of viscosity, where higher polymer concentration increases chain entanglement resistance to jet stretching.

The tip-to-collector distance and feed rate exhibit a comparatively smaller effect on the model output. Although less significant, the plot for flow rate shows a clear trend where higher values (red points) generally correspond to positive SHAP values, suggesting that increasing the flow rate has a tendency to increase the fiber diameter. This can be attributed to a greater volume of material being fed to the jet per unit of time, which can counteract the stretching forces to some extent. This ranking of feature importance–with voltage and concentration as primary drivers and distance and flow rate as secondary factors–is consistent with established electrospinning literature.

#### Hyperparameter tuning results for optimization algorithms

Table 5 summarizes the selected hyperparameter configurations for all optimization algorithms, as determined via grid search on the surrogate model using the development set.

**Table 5:** Summary of the best hyperparameter configurations for each optimization algorithm via grid search on the surrogate model using the entire 80% development set. Random search and brute-force grid search do not have tunable hyperparameters.

Optimizer	Best hyperparameters
Simulated annealing (SA)	Initial temperature = 200, cooling rate = 0.98, iterations per temp = 50, step size = 0.2
Genetic algorithm (GA)	Population size = 50, generations = 100, crossover rate = 0.8, mutation rate = 0.05, mutation scale = 0.2
Differential evolution (DE)	Population size = 70, generations = 170, crossover rate = 0.6, mutation factor = 0.6
Particle swarm optimization (PSO)	Number of particles = 50, $w_{\max } = 0.9$, $w_{\min } = 0.3$, $c_1 = 1.5$, $c_2 = 1.5$, max velocity = 0.3
Bayesian optimization (BO)	$n_\textrm{init} = 10$, $n_\textrm{iter} = 50$, acquisition function = EI, $\kappa = 2.5$, $\xi = 0.01$
Random search	N/A
Brute-force grid search	N/A

### Optimization performance

The performance of the seven optimization algorithms in identifying process parameters to achieve target nanofiber diameters is summarized in Table 6. Among all methods, Particle Swarm Optimization (PSO) demonstrated the highest inverse-design performance, achieving the lowest inverse design errors (RMSE=$3.626 \pm 1.302$ nm, MAE=$1.777 \pm 0.779$ nm) together with the highest coefficient of determination ($R^2=0.991 \pm 0.004$, 95% CI: 0.983–0.999). The narrow confidence intervals across all metrics indicate that PSO consistently identifies parameter sets that drive the surrogate-model output very close to the target diameter, with minimal run-to-run variability.

Bayesian Optimization (BO) also exhibited strong performance, yielding an RMSE of $4.769 \pm 1.556$ nm, an MAE of $2.502 \pm 0.988$ nm, and an $R^2$ value of $0.985 \pm 0.006$. Although slightly less accurate than PSO, BO maintained relatively tight confidence intervals, indicating stable convergence behavior across independent runs.

Brute-force grid search performed comparably well, achieving an RMSE of $6.184 \pm 2.106$ nm, an MAE of $2.858 \pm 1.334$ nm, and an $R^2$ of $0.974 \pm 0.011$. The relatively tight confidence intervals of grid search reflect its deterministic nature and further support its reliability, although its discretized search space limits its ability to match the continuous refinement achievable by PSO.

In contrast, Random Search exhibited a poor performance (RMSE = $21.439 \pm 5.620$ nm, MAE = $11.434 \pm 4.301$ nm, $R^2 = 0.699 \pm 0.084$), with wide confidence intervals highlighting substantial run-to-run variability and the inefficiency of unguided sampling in this nonlinear parameter space. Simulated Annealing and Differential Evolution also underperformed, with Simulated Annealing yielding an $R^2 = 0.384 \pm 0.100$ and Differential Evolution achieving an $R^2 = 0.708 \pm 0.083$, both accompanied by high prediction errors and broad confidence intervals. These results suggest a higher likelihood of convergence to suboptimal regions and greater variability across independent runs under the present surrogate-model and hyperparameter settings.

The Genetic Algorithm achieved moderate performance, with an RMSE of $15.977 \pm 5.193$ nm, an MAE of $7.275 \pm 3.445$ nm, and an $R^2 = 0.829 \pm 0.065$, although its wider confidence intervals indicate less consistent convergence compared to PSO and BO.

**Fig. 9:**
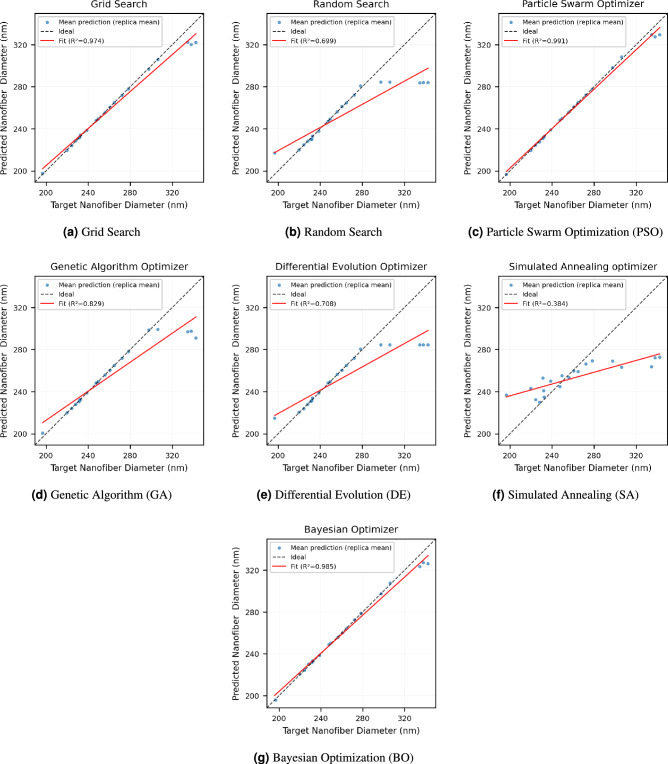
Optimization results for nanofiber diameter prediction using different inverse-design algorithms. Each subfigure shows the mean predicted diameter as a function of the target diameter for a given optimization method. Results are aggregated across five independent optimization replicas.

The plots in Fig. 9 visually corroborate these trends. In particular, the PSO replicates show nearly complete overlap across all target diameters, demonstrating not only small optimization error but also exceptional repeatability. The mean predicted diameters closely follow the target values, with only minor deviations at the extremes of the diameter range, where surrogate-model uncertainty is higher. Together, the quantitative metrics, confidence intervals, and replicate trajectories highlight the robustness and reliability of PSO relative to the other methods evaluated.

**Table 6:** Comparison of optimization methods on original candidate sets.

Method	Original candidates		
	RMSE	MAE	$R^2$
Particle swarm optimization	3.626 ± 1.302 [0.777, 5.971]	1.777 ± 0.779 [0.496, 3.503]	0.991 ± 0.004 [0.983, 0.999]
Bayesian optimization	4.769 ± 1.556 [0.967, 7.600]	2.502 ± 0.988 [0.831, 4.640]	0.985 ± 0.006 [0.972, 0.997]
Grid search	6.184 ± 2.106 [0.607, 9.862]	2.858 ± 1.334 [0.530, 5.766]	0.974 ± 0.011 [0.954, 0.999]
Genetic algorithm	15.977 ± 5.193 [2.259, 24.895]	7.275 ± 3.445 [1.688, 14.428]	0.829 ± 0.065 [0.701, 0.987]
Differential evolution	21.127 ± 5.592 [8.624, 31.710]	11.142 ± 4.259 [4.059, 19.604]	0.708 ± 0.083 [0.537, 0.873]
Random search	21.439 ± 5.620 [8.662, 32.157]	11.434 ± 4.301 [4.301, 20.047]	0.699 ± 0.084 [0.528, 0.843]
Simulated annealing optimization	30.818 ± 6.079 [18.626, 42.311]	21.412 ± 5.165 [12.415, 31.869]	0.384 ± 0.100 [0.151, 0.545]

### Optimizer run times

The computational efficiency of each optimization algorithm was evaluated by measuring the total run time required to complete the inverse design task. The results, summarized in Table 7, were obtained on an Apple MacBook (M1, 16 GB RAM) and are reported as mean ± standard deviation.

Random Search was the fastest method, with a mean run time of $0.0125 \pm 0.0049$ s, reflecting its simple, non-iterative sampling strategy. Simulated Annealing and Differential Evolution also exhibited low computational overhead, with run times of $0.0176 \pm 0.0124$ s and $0.0271 \pm 0.0090$ s, respectively.

Brute-force Grid Search required a longer execution time ($0.1195 \pm 0.0079$ s), as it systematically evaluates all candidate combinations within the predefined parameter grid. Particle Swarm Optimization showed moderate computational demand, completing the optimization in $0.1841 \pm 0.0275$ s, despite its superior accuracy and robustness.

Genetic Algorithm exhibited higher computational cost, with a mean run time of $0.4809 \pm 0.0445$ s, reflecting the overhead associated with population evolution across generations. Bayesian Optimization was the most computationally expensive method, requiring $3.2159 \pm 1.1580$ s due to the repeated construction and updating of its probabilistic surrogate model.

These results indicate that while all optimizers are computationally feasible for small-scale inverse design tasks, the choice of algorithm has a noticeable impact on total computation time, highlighting the trade-off between optimization accuracy and computational efficiency.

**Table 7:** Total optimizer run times (mean ± std) measured on an Apple MacBook (M1, 16 GB RAM).

Optimizer	Total run time (s) Mean ± Std
Random search	0.0125 ± 0.0049
Simulated annealing (SA)	0.0176 ± 0.0124
Differential evolution (DE)	0.0271 ± 0.0090
Grid search	0.1195 ± 0.0079
Particle swarm optimization (PSO)	0.1841 ± 0.0275
Genetic algorithm (GA)	0.4809 ± 0.0445
Bayesian optimization (BO)	3.2159 ± 1.1580

## Discussion, limitations, and future directions

This study successfully demonstrated a robust, data-driven framework for the inverse design of electrospun nanofibers, directly addressing the challenge of identifying optimal process parameters to achieve a specific fiber diameter. By moving beyond traditional forward modeling, our work provides a practical methodology to accelerate materials design and reduce the reliance on costly and iterative experimentation.

The selection of XGBoost as the surrogate model proved to be highly effective, achieving a testing R^2^ of 0.89. Its success stems from its ability to capture the complex, non-linear relationships between the four process parameters and the resulting fiber diameter. The high predictive accuracy of the surrogate is further bolstered by the SHAP analysis (Fig. 8), which confirmed that the model learned physically meaningful relationships. The findings that applied voltage and polymer concentration are the most influential parameters, and their respective negative and positive correlations with fiber diameter, align perfectly with the established principles of electrohydrodynamics and polymer jet physics. This interpretability is crucial, as it builds confidence that the surrogate is not merely a “black box” but a reliable digital twin of the experimental process.

The performance differences among the regression models can be attributed to the nonlinear and coupled relationships between the electrospinning parameters and the resulting nanofiber diameter. Tree-based ensemble methods such as XGBoost and Gradient Boosting outperform linear and distance-based models because they can capture higher-order interactions and complex, non-additive effects present in the experimental data. These methods build sequential learners that iteratively correct residual errors, allowing them to model curvature, thresholds, and parameter couplings that are inherent to electrospinning physics. In contrast, linear models assume additive and independent effects and therefore fail to represent the strong nonlinear dependencies between variables such as voltage, concentration, and feed rate, resulting in low explanatory power and poor generalization. Methods such as KNN and single decision trees also underperform because they are sensitive to data sparsity, noise, and boundary effects, which are amplified in a moderate-sized dataset with uneven coverage across the parameter space. Ensemble averaging mitigates these limitations, making XGBoost particularly effective by reducing variance, preventing overfitting, and enabling robust modeling of nonlinear relationships. These characteristics explain the clear performance gap observed across the regression models.

The central finding of our inverse design task is the exceptional performance of Particle Swarm Optimization (PSO), which achieved the highest inverse design accuracy among all evaluated methods, slightly surpassing even brute-force grid search. This result is significant for two key reasons. First, it highlights the effectiveness of metaheuristic search algorithms such as PSO in navigating a continuous parameter space. While brute-force grid search is restricted to a discrete set of predefined points, PSO can explore intermediate regions of the parameter space, enabling more precise minimization of the objective function. This capability is particularly important for fine-tuning manufacturing processes in which small parameter variations can lead to substantial changes in material properties. Second, PSO offers a favorable balance between optimization accuracy and computational efficiency. As shown by the measured run times, PSO requires substantially less computational effort than more complex methods such as Bayesian Optimization, while remaining scalable compared to exhaustive grid search approaches. If additional process parameters, such as humidity or temperature, were incorporated, the computational cost of grid search would increase exponentially, whereas the cost associated with PSO would grow more modestly, ensuring the continued practicality of the framework for higher-dimensional inverse design problems.

The differences in performance among the optimization algorithms can be understood by considering the structure of the surrogate-model error landscape and the search strategies inherent to each method. Algorithms such as Particle Swarm Optimization (PSO) and brute-force grid search perform well because they are able to reliably explore the continuous parameter space and converge toward globally optimal regions. PSO achieves this by combining global exploration with social learning and local refinement, allowing it to efficiently navigate the smooth but nonlinear error surface defined by the surrogate model. Brute-force grid search performs similarly well due to its exhaustive coverage of a discretized parameter space, although it lacks the fine-grained continuous refinement achievable by PSO.

Bayesian Optimization (BO) also demonstrates strong inverse-design performance by constructing a probabilistic model of the objective function and using an acquisition strategy to balance exploration and exploitation. This enables BO to focus sampling in promising regions of the parameter space, leading to high optimization accuracy with relatively few evaluations. However, the computational overhead associated with repeatedly updating the probabilistic surrogate limits its efficiency compared to PSO, particularly as the dimensionality of the search space increases. In contrast, methods such as Random Search and Simulated Annealing exhibit comparatively weaker performance in this study, because they rely heavily on unguided sampling or single-solution trajectories, which increases the likelihood of convergence to suboptimal regions or insufficient coverage of the parameter space.

Differential Evolution and the Genetic Algorithm show intermediate performance: while population-based strategies improve global exploration and enable escape from local minima, their convergence behavior is more sensitive to hyperparameter settings and often requires larger populations or more iterations to approach the stability achieved by PSO. These mechanism-level differences explain the observed variation in inverse-design accuracy, robustness, and computational efficiency across the evaluated optimization algorithms.

Despite the promising results, this study has several limitations that provide clear avenues for future research. First, the surrogate model’s predictive capabilities are inherently tied to the scope of the training data. The model is specific to the electrospinning of poly(vinyl alcohol) (PVA) with a particular molecular weight in distilled water and cannot be expected to generalize to other polymer-solvent systems without retraining on new, relevant experimental data. Given that obtaining sufficient new data for certain polymer systems can be challenging, advanced strategies like Transfer Learning or Domain Adaptation can be adopted as data-efficient methods to fine-tune the model learned on one polymer system to quickly adapt and generalize to a new polymer system. Achieving a truly generalized model that can predict across different polymer types will also require expanding the feature space to include intrinsic polymer descriptors, such as solubility parameters, or molecular weight distribution.

Second, the model does not account for ambient conditions such as temperature and humidity, which are known to significantly influence the electrospinning process by affecting solvent evaporation rates and jet stability. The exclusion of these variables represents a potential source of unmodeled variance and limits the direct transferability of the predicted “optimal” parameters to different laboratory environments. Therefore, future work must incorporate these ambient factors into the feature space to build a more comprehensive and transferable digital twin of the process.

Third, while the dataset of 96 experiments is systematically designed and well-structured, it is of moderate size for training complex machine learning models. Additionally, the dataset does not contain explicit experimental replicates that would quantify process repeatability under identical conditions. A larger dataset covering a wider range of parameter values with multiple replicates per condition could improve the model’s robustness and enable uncertainty quantification, particularly at the extremes of the processing window where data was sparser and for industrial applications requiring strict process control.

Building on these limitations, several promising directions for future research emerge. A critical next step is the experimental validation of the proposed framework, which would involve fabricating nanofibers using PSO-derived parameter sets and comparing the resulting diameters with the specified targets. Such validation would provide direct evidence of the framework’s practical utility.

An important extension of this work is the implementation of a closed-loop refinement system in which experimentally validated results are iteratively incorporated to update and retrain the surrogate model. This feedback mechanism would enhance model accuracy and adaptability over time, enabling the development of a self-improving design tool.

Future efforts should also aim to expand the framework by incorporating additional process parameters, such as ambient humidity, temperature, and solvent composition, as well as by predicting a broader set of fiber properties, including morphology, bead density, and mechanical characteristics. These extensions would enable multi-objective optimization and support the rational design of nanofibers with tailored functional properties.

In addition, the integration of constrained objective functions represents a relevant avenue for further development. While the current formulation minimizes the error between predicted and target diameters, it does not explicitly enforce constraints on the process parameters. Introducing constrained optimization, in which selected variables are fixed or restricted to discrete values, would better reflect real-world manufacturing limitations and improve the practical feasibility of the optimized solutions. This is particularly important for parameters that are inherently discrete or non-continuous but are currently treated as continuous variables during optimization.

Finally, the generalizability of the methodology should be assessed by applying and validating the framework across diverse polymer–solvent systems. Demonstrating successful transfer to other materials would highlight the broader applicability of the inverse design approach and its potential as a general tool for electrospinning process optimization.

## Conclusion

This study developed and validated a robust data-driven inverse design framework for electrospun nanofiber fabrication, overcoming the limitations of traditional trial-and-error methods and forward modeling approaches. By integrating a high-fidelity surrogate model with advanced optimization techniques, the framework provides an efficient and practical strategy to guide process parameters toward achieving targeted nanofiber diameters.

Among eleven regression models evaluated, Extreme Gradient Boosting (XGBoost) consistently delivered the highest predictive accuracy (testing R^2^ = 0.890), reliably capturing the complex, nonlinear relationship between process parameters and fiber diameter. SHAP analysis further confirmed the model’s physical plausibility, identifying applied voltage and solution concentration as the most influential factors.

In the inverse design phase, Particle Swarm Optimization (PSO) achieved exceptional performance (R^2^ = 0.991, MAE = 1.77 nm), slightly surpassing brute-force grid search. This demonstrates that metaheuristic algorithms can offer both high accuracy and computational efficiency for solving complex inverse design problems.

Beyond electrospun nanofibers, this framework has broad potential for predictive and prescriptive materials manufacturing. The synergistic combination of XGBoost and PSO provides a powerful strategy for material design and property control, with potential applications in chemical reaction optimization, novel material development in petroleum engineering, and molecular exploration in drug discovery.

Future work will focus on experimental validation of the optimized parameters, implementation of a closed-loop refinement system, and expansion of the framework to incorporate additional process parameters and fiber properties, enabling multi-objective and constrained-objective design and broader applicability across materials science.

## Data Availability

The training data used in this study are available from study of Ziabari et al.^30^. The optimization log files (containing detailed results for each algorithm) are available on the corresponding GitHub repository referenced in the Code Availability section.

## Code availability

he underlying code for this study is publicly available on GitHub at https://github.com/taltechloc/cogni-e-spin-inverse-design.git.
